# The Influence of the Crown-Implant Ratio on the Crestal Bone Level and Implant Secondary Stability: 36-Month Clinical Study

**DOI:** 10.1155/2018/4246874

**Published:** 2018-05-16

**Authors:** Jakub Hadzik, Maciej Krawiec, Konstanty Sławecki, Christiane Kunert-Keil, Marzena Dominiak, Tomasz Gedrange

**Affiliations:** ^1^Department of Dental Surgery, Wroclaw Medical University, ul. Krakowska 26, 50-425 Wrocław, Poland; ^2^Department of Oral Implantology, Wroclaw Medical University, ul. Krakowska 26, 50-425 Wrocław, Poland; ^3^Department of Orthodontics, Technische Universität Dresden, Carl Gustav Carus Campus, Fetscherstr. 74, 01307 Dresden, Germany

## Abstract

**Introduction:**

When the era of dental implantology began, the pioneers defined some gold standards used in dental prosthetics treatment for implant-supported restorations. Referring to traditional prosthetics, it was taken for granted that the length of an implant placed in the alveolar bone (the equivalent of the root) should exceed the length of the superstructure.

**Aim of the Study:**

The aim of the study was to determine whether implant length and the crown-to-implant (*C*/*I*) ratio influence implant stability and the loss of the surrounding marginal bone and whether short implants can be used instead of sinus augmentation procedures.

**Material and Methods:**

The patients participating in the study (*n* = 30) had one single tooth implant, a short (OsseoSpeed™ L6 Ø4 mm, Implants) or a regular implant (OsseoSpeed L11 and L13 Ø4 mm, DENTSPLY Implants), placed in the maxilla. The evaluation was based on clinical and radiological examination. The crown-to-implant ratio was determined by dividing the length of the crown together with the abutment by the length of the implant placed crestally. Mean crown-to-implant ratios were calculated separately for each group and its correlation with the MBL (marginal bone loss) and stability was assessed. The authors compared the correlation between the *C*/*I* ratio values, MBL, and secondary implant stability.

**Results:**

Positive results in terms of primary and secondary stability were achieved with both (short and conventional) implants. The MBL was low for short and conventional implants being 0.34 ± 0.24 mm and 0.22 ± 0.46 mm, respectively. No significant correlation was found between the *C*/*I* ratio and secondary stability as well as the *C*/*I* ratio and the marginal bone loss.

**Conclusions:**

Short implants can be successfully used to support single crowns. The study has revealed no significant differences in the clinical performance of prosthetic restorations supported by short implants. Clinical trial registration number is NCT03471000.

## 1. Introduction

The crown-to-root (*C*/*R*) ratio is commonly used by dental clinicians to qualify a tooth for a fixed dental crown. It is believed that a proper *C*/*R* is one of the key factors in achieving a long-term prognosis in prosthetic rehabilitation [1–3]. The importance of a proper *C*/*R* ratio may be explained by the biomechanical concept of a class I lever, so when a disproportionate *C*/*R* ratio occurs, the periodontium is more susceptible to injury due to heavy occlusal forces. This phenomenon was studied, for example, by McGuire and Nunn in a prospective study on predicting tooth loss for periodontal patients [4]. There are no strict guidelines for a *C*/*R* ratio, but when a periodontium is healthy the optimal *C*/*R* ratio for a fixed crown is considered 1 : 2 or less [1, 5].

When the era of dental implantology began, clinicians started using certain guidelines associated with natural teeth for the implant-supported fixed crowns. It was taken for granted that the length of an implant placed in the alveolar bone (the equivalent of the root) part should exceed the length of the superstructure.

Many studies have shown that the success of implant osseointegration is considerably dependent on its surface and it has been proven that osteoblastic cells adhere more quickly to rough surfaces [6–8]. Many methods for increasing the dental implant roughness were described; one of them is the the sandblasted and acid-etched surface created by the combination of sand-blasting and acid-etching is the most relevant and most commonly used method, and its significance has been documented in numerous studies [9–11].

Before implant surface modifications were widely recognized in the literature, in case of insufficient bone volume, augmentation procedures had been the only solution to ensure sufficient bone volume [12]. In the maxilla, in cases with vertical dimension deficiency, the augmentation in the maxillary sinus prior to the planned implantation is often necessary to obtain sufficient bone volume to stabilize a dental implant. Many biomaterials (including autografts, allografts, xenografts, and alloplasts) may be successfully used in these techniques, but sinus lift procedures feature the risk of complications, such as the perforation of the Schneiderian membrane (7–30% depending on the used technique and instruments) [13–15].

The indisputable progress and improvement of the implant surface enabled implant length reduction while still maintaining proper stability and functionality. Short implants could be used in cases where traditional implants preceded by the grafting procedure were the only solution. Furthermore, the implant surface modification also made it possible to stop following the guidelines used in traditional prosthetics. The barrier to maintaining a proper crown-to-implant (*C*/*I*) ratio was exceeded. A considerable number of studies addressed the issue of implant length as a predictor of implant survival, but they achieved inconclusive results. However, it has been pointed out that the excessive *C*/*I* ratio could impair long-term implant survival [16].

On the other hand, the recent literature indicates very promising results and argues that short implants may safely replace regular implants; however, due to their sophisticated surface, short implants remain stable when loaded with a crown longer than the implant itself [17–19].

The aim of the study was to check whether the crown-implant ratio influences the secondary implant stability and the marginal bone level [MBL] in implants loaded with single nonsplinted crowns. It was also assessed whether the use of increased *C*/*I* ratios for short implants would be as successful as for long implants proceeded by maxillary sinus augmentation with a xenograft.

## 2. Material and Methods

For the purpose of unifying the nomenclature in the manuscript, authors use the word superstructure for the prosthetic crown with the abutment.

### 2.1. Experimental Design

This prospective study was conducted based on clinical and radiographic examination. The study protocol was approved by the local ethical commission (Bioethics Committee at Wroclaw Medical University, approval number KB 427/201). All patients gave two written consents: the first was general consent to have dental implants placed, and the other consent involved the participation in the study. The study has been conducted in full compliance with the Declaration of Helsinki. The primary protocol of the study assumed a larger group of patients; however, only 30 patients were included in this long follow-up period. Other patients because of long evaluation period resigned from participation in the project; others because of poor compliance were excluded from the project.

The evaluation in this study group of patients incorporated 30 adults (10 males, 20 females), with a mean age of 45.5 years, who had DENTSPLY implants placed at the Department of Dental Surgery at Wroclaw Medical University. The patients who met the inclusion criteria were divided at random (by drawing lots) into two groups according to the method of treatment provided.

Group 1 (G1; *n* = 15 patients) had conventional dental implants (OsseoSpeed L11 Ø4 mm and L13 Ø4 mm) [DENTSPLY Implants, Waltham, MA, USA] placed, preceded by the sinus lift procedure from a lateral window approach with the application of the xenogeneic bone graft Geistlich Bio-Oss® [Geistlich AG, Wolhusen, Switzerland]. The lateral window approach sinus lift surgery was performed 6 weeks prior to the implant placement by the same surgeon.

Group 2 (G2; *n* = 15 patients) had short implants (OsseoSpeed L6 mm Ø4 mm) [DENTSPLY Implants, Waltham, MA, USA] placed without sinus lift and augmentation procedure.

### 2.2. Inclusion and Exclusion Criteria

Nonsmoking patients with no systemic or local diseases were qualified.

Additional inclusion criteria were as follows:

- Minimal apicocoronal height of the alveolar ridge of 6 mm in the region of the implant insertion in the presurgical qualification
- Minimal width of the alveolar ridge of 6-7 mm in the region of interest
- HKT (height of the keratinized tissue) higher than 2 mm
- API ≤ 35 (Approximal Plaque Index)
- PI ≤ 25. (Plaque Index)
- Bone Type III or D2 were included in the study
- No graft procedures in the area of interest

In both groups, D2 (Misch) was the radiologically and clinically assessed bone density based on presurgery CT scans and intrasurgery clinical evaluation. The surgical procedure was performed under the same conditions and by the same medical team with induced local anesthesia. All patients were instructed to rinse their mouths with 0.12% chlorhexidine solution (twice a day until suture removal) and to take the prescribed antibiotics and analgesics (Augmentin 1,0 in tabl. One dose at one hour before the surgery and then 5 days after implant placement 1,0 g every 12 hours). In addition patients in Group 1 where the sinus floor was elevated received additional antibiotic therapy when the surgery was performed (Augmentin 1,0 in tabl. One dose at one hour before the surgery and then 5 days after implant placement 1,0 g every 12 hours). Nonresorbable sutures were removed 7–14 days after the implant placement. In all cases, final restorations were manufactured and cemented with resin based semipermanent cement 6 months after implant placement. All implants in this study were loaded with single nonsplinted crowns.

CBCT (Cone Beam Computed Tomography) [Galileos® D3437, Sirona Dental, Germany] and RVG [Visualix® eHD, Gendex Dental Systems, USA] were taken for each implant analyzed and measured to assess the crown-implant ratio. The initial CBCT and RVG taken immediately after the implant placement (T0) and CBCT and RVG radiographs taken after 36 month (T1) were used to assess the marginal bone level changes. The loss of the marginal bone was measured based on the CBCT image and using a standard RVG periapical X-ray done with the use of a straight angle technique with a positioner. The CBCT image offers transrectal views so the measurement can be made around the implant. On the periapical X-ray, the bone level was measured on the mesial and distal site of the implant and the mean values were calculated. The measuring points on CBCT were located around the implant (4 points around each mesial, distal, buccal, and palatal) and the mean values were calculated. To indicate the value in millimeters, in each case the radiological measurement was calibrated with the previously known length of the implant. Then the mean value of the measurements from both CBCT and RVG was calculated and these mean values were presented in the manuscript.

For Periotest®, measured in PVT Periotest Values [Periotest Classic, Medizintechnik Gulden, Germany] examination was performed to assess secondary implant stability after 36 months. In all cases, the Periotest evaluation was conducted in the same manner. Each implant was evaluated at 4 different location points, each with a different direction of the excitation: 2 points at the buccal (45 degrees from the mesiobuccal direction, 90 degrees from the buccal direction, both at the half the height of the supragingival part of the crown) and similarly on the palate, each excitation place was evaluated 3 times. The mean was calculated for all evaluation points for each implant.

**Figure 1:**
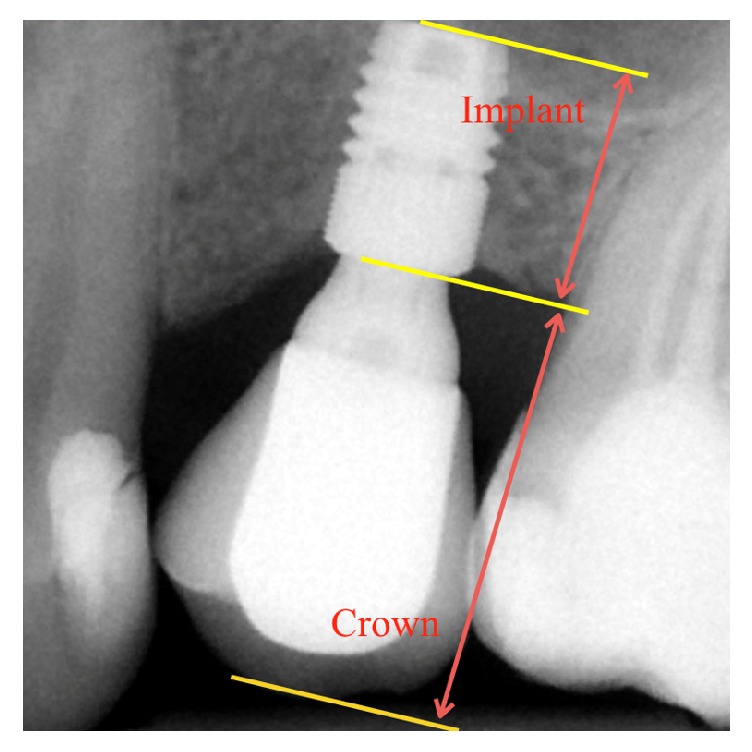
Periapical digital radiograph of a short implant (Astra Tech implant system™ OsseoSpeed TX 4.0 S; Ø4 mm, 6 mm long). The *C*/*I* ratio measurement method is presented. Radiological status 36 months after implant placement.

**Figure 2:**
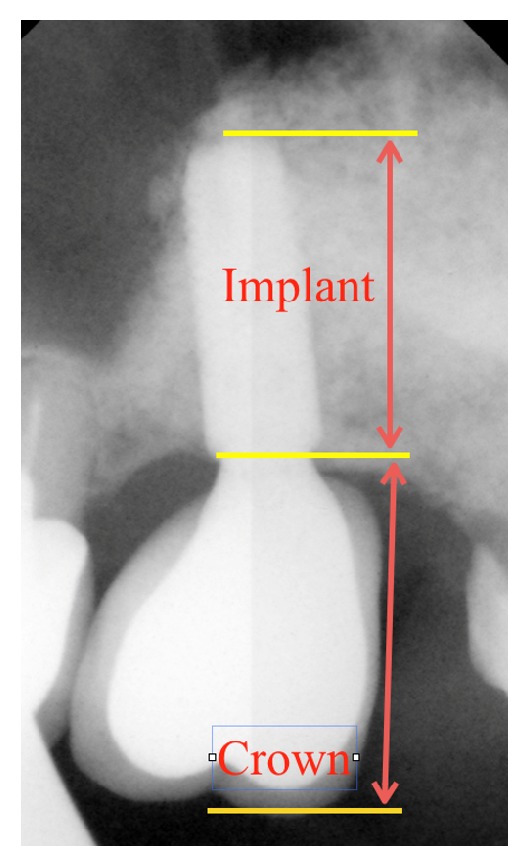
Periapical digital radiograph of a regular implant (Astra Tech implant system OsseoSpeed TX 4.0 S; Ø4 mm, 11 mm long). The *C*/*I* ratio measurement method is presented. Radiological status 36 months after implant placement.

The crown-to-implant ratio was determined by dividing the length of the superstructure (crown and the abutment) by the length of the implant that was placed crestally (Figures 1 and 2). Mean crown-to-implant ratios were calculated separately for each group and its correlation with the MBL (MBL = marginal bone loss) and implant stability was evaluated. The authors compared the correlation between the range of *C*/*I* ratio values, the MBL, and secondary implant stability, respectively.

### 2.3. Statistical Analysis

The statistical analysis was performed using GraphPad Prism 6 software [GraphPad Software, Inc., USA]. Spearman's rho test was used to measure correlation. All data were given as means ± standard deviation (SD). *P* < 0.05 was considered statistically significant.

## 3. Results

The evaluation of implant stability with Periotest after 36 months (T1) yielded good results of secondary implant stability in both groups (G1 and G2: 0.93 ± 3.39 PTV and 1.0 ± 2.7 PTV). The marginal bone level loss was low and similar in both groups (G1 and G2: 0.22 ± 0.46 mm and 0.34 ± 0.24 mm). No significant difference in the MBL between short and regular implants was found (Table 1).

The average *C*/*I* ratio in G1 was 1.063 and in G2 1.69 (Table 2). No significant correlation between the *C*/*I* ratio and the secondary stability was found as well as for the *C*/*I* ratio and the marginal bone loss (Table 3).

## 4. Discussion

There is still some controversy over the definition of a short implant. According to Tawil and Younan, an implant of ≤10 mm is considered short [20], whereas Nisand and Renouard define the one with a designed intrabony length of ≤8 mm as short and a device with a designed intrabony length of ≤5 mm as extra short [21]. In our study, the implants with a length of 11 or 13 mm were considered regular, whereas 6 mm dental implants were considered short. The study only used single crown restorations for both G1 and G2. Splinting the crowns when examining the impact of the *C*/*I* ratio would change the distribution of forces and, consequently, the results would be disturbed. The determination of the marginal bone level (MBL) was based on radiographic measurements after 36 months. The marginal bone level loss was low and similar in both tested groups. No significant difference in the MBL between short and conventional implants was found, as well as no correlation between the MBL and the *C*/*I* ratio. Furthermore, the correlation between the *C*/*I* and implant stability was reported not to be statistically significant. These results may correspond with a majority of the *C*/*I* ratio studies arguing that the crown-to-root ratio guidelines associated with natural teeth should not be directly applied when planning implant-supported single tooth restoration.

The recent literature shows that the crown-to-implant ratio has no major impact on the clinical performance of implants and may be successfully applied [19, 22–24]. In the systematic review by Blanes it was found that the *C*/*I* ratios of implant-supported reconstructions do not influence peri-implant crestal bone loss [22]. Mangano et al. studied 68 short dental implants over a period of 5 years with different *C*/*I* ratios. No significant differences were found in the survival rate, prevalence of biological complications, and prosthetic complications between the groups with *C*/*I* ≥ 2 and *C*/*I* < 2 [24]. Those findings correspond to ours. We have found no correlation between the *C*/*I* ratio and the MBL. Schulte et al. analyzed retrospectively 889 single tooth implants from 294 patients and put forward that the crown-to-root ratio guidelines associated with natural teeth should not be applied to a potential implant site or an existing implant restoration [19]. Schneider et al. in a 5-year retrospective investigation demonstrated that the *C*/*I* ratio did not influence the clinical performance of implants supporting single crown restorations in the posterior segments of the jaw [23]. However, a significant negative association between the crown-implant ratio and the marginal bone loss was described in the literature as well. The systematic review by Garaicoa-Pazmiño et al. revealed that the *C*/*I* ratio of implant-supported restorations has an effect on the peri-implant marginal bone level [25]. Malchiodi et al. achieved similar results in his study. These authors analyzed 259 short dental implants in 136 patients over a period of 36 months. They observed a significant correlation between the clinical *C*/*I* ratio and the crestal bone loss. The peri-implant bone loss was significantly increased for implants with the *C*/*I* ≥ 2 [26]. The study by Nunes et al. evaluated 118 implants from 59 patients, where 30 implants presented the *C*/*I* ≤ 2 and 88 implants the *C*/*I* > 2. The authors revealed a weak inverse but insignificant correlation between the *C*/*I* ratio and the MBL [27]. They concluded that implant-supported fixed prostheses with the *C*/*I* ratio of >2 have a negative impact on the MBL. Anitua et al. managed to demonstrate that the use of a cantilever for prosthetic rehabilitation had a negative impact on the MBL. When the cantilever was used, the MBL was increased considerably by 238%. In contrast, when the cantilever was not used, the MBL is independent from the *C*/*I* ratio [28]. Using the finite element method, it was demonstrated that the stress concentration and stress distribution increase with the height of the crown [29, 30]. As the *C*/*I* ratio increased twice, the von Mises stresses rose by about 47%. At the *C*/*I* ratio of 2/1, the highest stresses were observed around the implant neck [29].

The improper placement of a dental implant and, consequently, the improper direction of the occlusive forces may lead to increased stress and strain distribution on the bone around the dental implants; therefore, the marginal bone loss and recession of the soft tissues may occur [31–33]. However, the authors of the study gave the proper location of the implants careful thought, and thus none of these occurred.

Authors have presented only one scenario for the use of short implants, which seems to be the very common clinical situation. Maxillary molars are most often prematurely lost in maxilla. Due to loss of bone volume and maxillary sinus expansion, bone conditions often do not allow for placing regular length implants. This is a clinically important since here short implants are an alternative to regenerative treatment such as sinus lift procedures that can feature the risk of complications. According to Oikarinen et al. who studied over 400 patients the available bone height in the posterior maxilla in 38% of cases is at least 6 mm [34]; this is just enough bone volume to consider short dental implant without supportive regenerative treatment. The loading with nonsplinted crowns is also groundbreaking and clinically relevant since many previous studies like Cannizzaro et al. [35], Esposito et al. [36], and Pistilli et al. [37] evaluate splinted crowns in similar conditions; however, Guljé et al. [38] and Thoma et al. [39] have presented good clinical outcome with nonsplinted short implants in maxilla.

Of course, the lack of single tooth in the maxilla is not the only indication for the short implants. They can successfully be used to avoid regeneration procedures in a atrophic mandible; among many studies over this issue also our research group Hadzik et al. presented successful application of short dental implants to replace two missing molars in atrophic mandible [40]. The literature shows many more fine examples of common clinical situations where short implants are used to avoid less predictable and difficult regenerative treatments combined with regular implants. These implants have been described by Esposito et al. as effective in rehabilitation of fully edentulous atrophic maxilla [41]; Maló et al. has presented a short-term outcome study with successful immediate loading of short implants in edentulous maxilla using all-on-4 concept [42].

## 5. Conclusion

In conclusion, due to the fact that no negative impact of the lowered *C*/*I* ratio on the marginal bone level and implant stability was found, we came to the conclusion that short implants may be successfully used to support a single crown. The clinical performance of short implants is comparable to regular implants. Both treatment modalities can be considered in the atrophic posterior maxilla; however short implants may be more favourable regarding short-term patient morbidity.

## Figures and Tables

**Table 1:** Marginal bone level loss. T0 compared to T1. Mean value ± SD.

	Group 1	Group 2	Wilcoxon test
T0 versus T1	0.22 ± 0.46 mm	0.34 ± 0.24 mm	*P* = 0.1229

**Table 2:** Correlations between *C*/*I* ratio: secondary stability and *C*/*I* ratio: marginal bone level loss.

Correlation	*C*/*I* ratio: secondary stability	*C*/*I* ratio: marginal bone level loss
*r*	−0.04	0.32
Test	Spearman	Spearman
Significance	No	No

**Table 3:** Crown-implant ratio (*C*/*I*) after 36 months (T1).

Group 2	*C*/*I* Ratio	Group 1	*C*/*I* Ratio
min	1.36	min	0.68
max	1.97	max	1.65
Mean	1.679	Mean	1.063
SD	0.2129	SD	0.293
Median	1.69	Median	1.05

## Data Availability

The authors declare that they are in possession of complete data on the basis of which the results presented in the manuscript have been developed. The authors will make the data available to interested parties if necessary.
